# Polygraphic Results in High-Risk Infants Aged Under 3 Months

**DOI:** 10.3390/clockssleep7030042

**Published:** 2025-08-12

**Authors:** Daniel Zenteno, Gerardo Torres-Puebla, Camila Sánchez, Rocío Gutiérrez, María José Elso, Pablo E. Brockmann

**Affiliations:** 1 Department of Pediatrics, Faculty of Medicine, University of Concepción, Concepcion 4070409, Chilecam.rox.san.fer@gmail.com (C.S.); rgutierrez2018@udec.cl (R.G.); 2 Mechanical Ventilation and Sleep Unit, Pediatric Service, Guillermo Grant Benavente Hospital, Concepcion 4070038, Chile; klgo.gerardo.torres@gmail.com; 3 Department of Medical Specialties, Faculty of Medicine, University of Concepción, Concepcion 4070409, Chile; mjelso@gmail.com; 4 Department of Pediatric Pulmonology, School of Medicine, Pontificia Universidad Católica de Chile, Lira 85, Santiago 8320165, Chile

**Keywords:** infant, apnea, sleep-disordered breathing

## Abstract

This study described and analyzed the results of cardiorespiratory polygraphic studies in infants under three months who were hospitalized and monitored due to suspected apneas. Methods: Cross-sectional study. Patients aged <3 months hospitalized from 2011 to 2023 were included. All were referred for suspected apneas, and cardiorespiratory polygraphies (PG) were conducted simultaneous to non-invasive monitoring. Demographic, PG, and diagnostic variables were recorded. PG values were obtained and compared between diagnostic groups. Association was evaluated according to diagnosis, prematurity, presence, and alteration type with Kruskal–Wallis, Wilcoxon, and Fisher tests. Association between quantitative variables was assessed with Spearman’s rho and the presence of alteration with binomial logistic regression. Analysis was performed with Jamovi v.2.3, and statistical significance was defined as *p* < 0.05. Results: A total of 155 studies were included. Median age was 41.0 days (IQR 22.0–59.0), median gestational age was 38 weeks (IQR 32.0–42.0), and 52.3% were premature. Diagnosis: brief resolved unexplained events (BRUE) (58.1%), apnea of prematurity (27.1%), hypotonic syndrome (7.1%), laryngomalacia (LGM) (3.9%), and craniofacial alterations (CFA) (3.9%). Altered results in 21.9% polygraphies: 44.1% with AHI ≧ 5/h and 20.6% with SpO_2_ ≦ 90% in >5% of the record. CFA and LGM patients had a higher risk of an altered polygraph than those with apnea of prematurity (OR 21.3/8.5) and BRUE (OR 35.9/14.3), respectively. Conclusions: Infants under three months of age referred for apnea showed often abnormal polygraphic indices, showing significant differences between diagnostic groups. Performance of sleep studies in these groups was feasible and allowed to confirm the presence of apneas and their level of severity. Particular attention should be considered in children with CFA and LMG, since their risk is significantly higher. Age-specific apnea patterns seem to be of interest, as this may possibly lead to future consequences.

## 1. Introduction

Sleep medicine has advanced significantly in the last decade, especially for pediatric patients, in whom sleep studies can determine relevant therapeutic conducts [1]. Newborns and young infants are characterized by often presenting symptoms that are difficult to evaluate and unspecific; therefore, a complete history, exhaustive physical examination, and, eventually, complementary studies can be essential for a diagnosis [2]. Polysomnography (PSG) has emerged as the gold standard for assessing sleep problems and sleep-disordered breathing in children.

In this age group, apneas constitute a challenge for health teams due to their etiological diversity and the high stress that they can generate in their parents or caregivers. Apneas may or may not be clinical evident. In neonates and newborns, apneas are often diagnosed by the presence of cyanosis or respiratory pauses. While there are respiratory patterns like periodic breathing that are often normal and physiological at this age, there may be also the presence of central and obstructive apneas that are considered to be abnormal. Many patients are considered at risk and require more detailed evaluation [2,3]. Therefore, implementing studies to assess the existence of a cardiorespiratory disorder during sleep is necessary for this group of patients [4,5]. Furthermore, circadian rhythm development may be affected in infants who exhibit sleep apneas and respiratory events.

While it is true that a thorough clinical assessment may lead to a proper diagnosis, sleep studies are often crucial in diagnosing underlying apneas that may not be otherwise identifiable. Cardiorespiratory polygraphy (PG) has been shown to have good diagnostic performance in pediatric patients, allowing its recommendation in both the hospital and home settings [6,7].

Recent studies suggest that evaluating children under three months of age using PG would provide clinically useful information. A recently published Chilean consensus proposed specific reference values for sleep studies at this age [4,8].

In some infants, especially premature and newborn, a pattern suggestive of respiratory immaturity has been described where central apneas are more frequent and periodic breathing includes a more significant percentage of sleep time, which is attributed to an alteration in respiratory control mediated by central and peripheral chemoreceptors [9]. Therefore, current sleep study interpretation is sometimes difficult at this age. Furthermore, there seems to be a lack of studies addressing polysomnographic and polygraphic findings in newborns and small toddlers.

Few centers use PG for sleep evaluation in children under three months of age and in newborns and infants aged <3 months at high risk of sleep apnea. The objective of the present study is to describe the results of PG in a court of children under three months of age with suspected apnea, and to compare the PG findings between diagnostic groups.

## 2. Results

**Figure 1:**
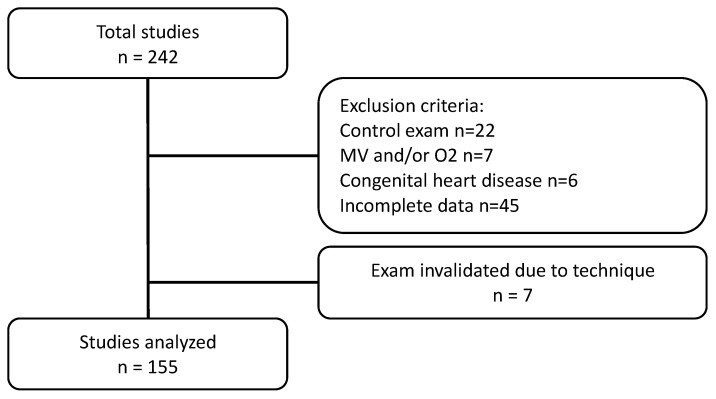
Eligibility criteria flowchart.

**Figure 2:**
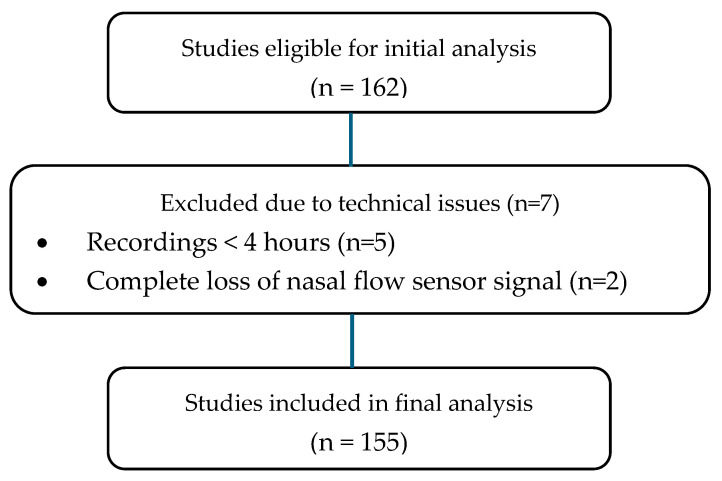
Detailed reasons for study exclusions.

During the study period, *n* = 242 PGs were performed on children aged <3 months hospitalized with suspected apneas; *n* = 80 were excluded (see Figure 1). Of the remaining 162, 7 were excluded due to the recording not being acceptable for interpretation; of them, 5 were due to recording time being less than 4 h and 2 due to loss of flow sensor (Figure 2).

**Figure 3:**
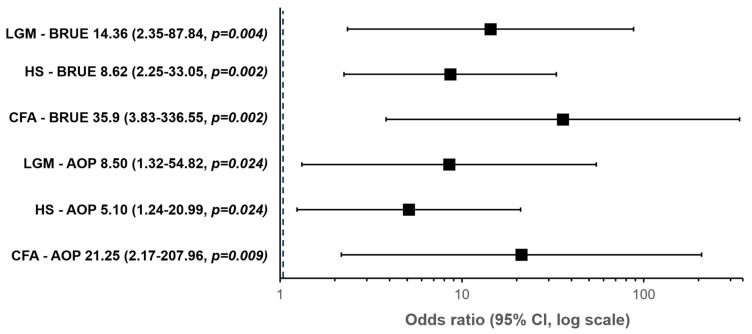
CFA: Craniofacial alterations; AOP: apnea of prematurity; LGM: laryngomalacia; HS: hypotonic syndrome.

Of the final *n* = 155 patients included, median age was 41 days (IQR 22.0–59.0); 63.9% were boys. The median gestational age (GA) was 38 weeks (IQR 32.0–42.0), and 52.3% had a history of prematurity. Regarding the diagnoses, 58.1% (*n* = 90) corresponded to high-risk BRUE, 27.1% (*n* = 42) apnea of prematurity (AOP), 7.1% (*n* = 11) hypotonic syndrome, 3.9% (*n* = 6) laryngomalacia (LGM), and 3.9% (*n* = 6) to craniofacial alterations (CFA) (Figure 3).

The median of total sleep duration was 9.6 h (IQR 8.8–10.3) with a validated artifact-free time of 6.7 h (IQR 5.9–10.3), observing a median AHI of 0.5 per hour (IQR 0.1–1.7), obstructive apnea index of 0.35 per hour (IQR 0.0–0.93), saturation of 97% (IQR 96.0–98.0) and minimum saturation of 85% (IQR 79.5–87.5). (Table 1 and Table S1).

**Figure 4:**
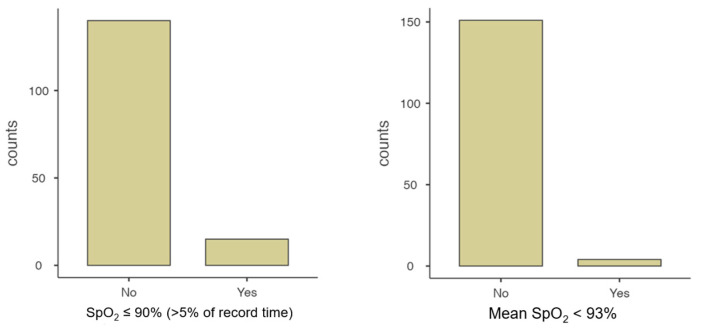
SpO_2_: Time spent below mean SpO_2_ < 93%, and SpO_2_ ≤ 90% (>5% of record time).

Overall, 21.9% (*n* = 34) of the polygraphies were abnormal; more than one diagnostic criterion coexisted in 11 (32.4%) studies. Thus, three diagnostic criteria were observed in 4 patients, two in 7 patients, and only one criterion in 23 patients. It was found that 50.0% (*n* = 19) of the study sample showed an AHI ≥ 5/h, 39.4% (*n* = 15) presented average SpO_2_ ≤ 90% in more than 5% of the record, and 23.7% (*n* = 9) had a CAI ≥ 1/h with average SpO_2_ ≤ 80% (Table 2 and Figure 4).

**Figure 5:**
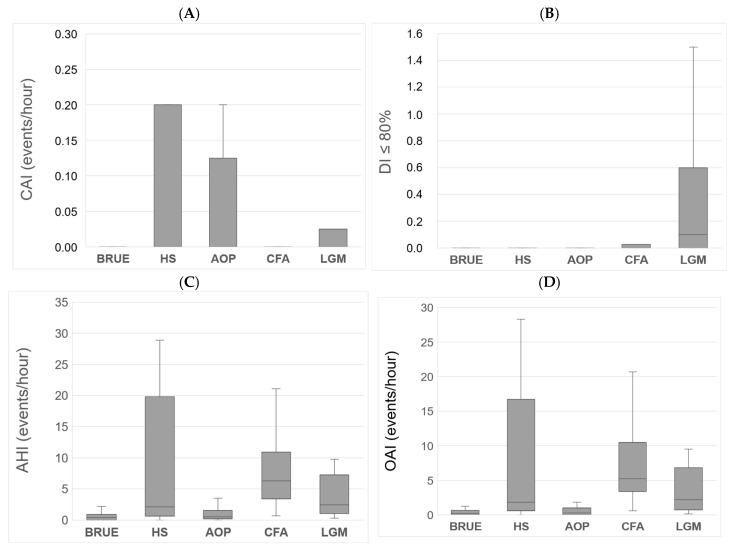
Polygraphic variables compared according to pathology. Respiratory indices are expressed in events/hours. The results are expressed in medians and IQR; AHI: apnea–hypopnea index; MOAHI: obstructive and mixed apnea–hypopnea index; CAI: central apnea index; DI < 80%: desaturation index less than 80%. (**A**) CAI according to each pathology; (**B**) DI ≧ 80% according to each pathology; (**C**) AHI according to each pathology; (**D**) OAI according to each pathology.

When the polygraphic variables were compared according to each pathology, there were differences in AHI (*p* < 0.001), MOAHI (*p* < 0.001), OAI (*p* < 0.001), SpO_2_ < 80% (*p* < 0.001), and DI ≤ 80 (*p* = 0.003). Those patients with CFA had a higher AHI compared to those with AOP (*p* = 0.021) and BRUE (*p* = 0.006), at the expense of a higher MOAHI (*p* = 0.006 and *p* = 0.003) and OAI (*p* = 0.009 and *p* = 0.002). Additionally, those with LGM had a higher DI ≤ 80 (*p* = 0.002) than those with BRUE (Figure 5).

When comparing premature and full-term children, it was observed that the former showed greater periodic respiration (*p* < 0.001) and lower minimum saturation (*p* < 0.001).

Patients with CFA (OR 21.25 *p* = 0.009), LGM (OR 8.5 *p* = 0.024), and hypotonic syndrome (HS) (OR 5.1 *p* = 0.024) have a higher risk of presenting PG alterations compared to patients with AOP. Likewise, patients with ACF (OR 35.9 *p* = 0.002), LGM (OR 14.4 *p* = 0.004), and SH (OR 8.6 *p* = 0.002) have a higher risk of presenting PG alterations compared to patients with BRUE (Table 3).

## 3. Discussion

This study showed frequent abnormal PG indices in children aged <3 months who were hospitalized and monitored simultaneously. It was observed that patients with CFA and LGM have a greater risk of presenting an altered study compared to other diagnoses, a situation that the treating teams must consider in order to assume specific behaviors for their clinical confrontation.

In this sample, 96% of the studies were considered valid, even higher than those published in other studies that include infants and older children [5,7,12,13], which means that only 4% of patients had to repeat the exam to provide interpretable results. From the non-validated studies, there were five studies with a duration of less than 4 h and two in which there was loss of the flow sensor or saturometer. Hence, feasibility of PG studies in children aged <3 months seems to be an attractive diagnostic tool for clinicians.

The primary diagnosis was high-risk BRUE, likely because of a higher incidence of this clinical entity. It is relevant to emphasize that those infants younger than two months or preterm newborns under 32 weeks with a corrected gestational age less than 45 weeks are considered at risk, a common condition in the group recruited in this study [3]. They are hospitalized to be monitored, and specific treatable causes are determined to avoid associated complications. Some authors have proposed that the highest risk age should include up to three months since it covers nearly 70% of apneas in infants, especially concerning the risk linked to respiratory infections, their recurrence, and their severity [14].

It is widely described that the primary diagnostic evaluation in this group of minors is an exhaustive clinical history and physical examination; however, when they are considered at risk, they should be studied to rule out infectious, cardiac, and metabolic causes, along with other evaluations on an individualized basis and based on the clinical suspicion of a particular pathology (e.g., pulmonary respiratory disorders, sleep disorders, neurological disease, inborn errors of metabolism, abuse, etc.) [11]. Recently published studies suggest increasingly limiting some routine examinations and incorporating studies that allow us to objectively diagnose apneas or hypoventilation to detect severe cases requiring early and appropriate intervention, avoiding potential complications [15]. Although polysomnography is the gold standard for diagnosing sleep-disordered breathing, polygraphy is a good alternative that can be performed on a hospitalized and monitored patient, which is an advantage in these cases. Our group published an article on PG in young infants with fewer studies, in which 33% corresponded to a diagnosis of major ALTE, which currently corresponds to high-risk BRUE [5].

Of all the PGs performed, 78.1% were normal, which agrees with the literature and establishes that most cases constitute single episodes and rarely present complications during sleep [11].

In the abnormal studies, the most frequent alteration was AHI ≥ 5/h (50%), mainly due to obstructive events; however, the coexistence of altered variables was also common (32.5%) and included findings suggestive of both intermittent and persistent hypoxemia. Central apneas were observed mainly in premature babies of lower gestational ages and were attributed to the immaturity of the respiratory control center. In previous works, we have demonstrated age-specific changes in central apneas [4]. In a follow-up study, central apneas decreased in the same subjects in relation to age, i.e., from newborns to three months of age [4]. This may suppose a maturation of several respiratory processes and also biological rhythms.

One of the most interesting results of this article is the evidently more significant alteration of the studies in patients with craniofacial alterations and laryngomalacia, compared with other diagnostic groups. Specifically, CFA are 35.9 and 21.3 times more likely to present with an abnormal PG than those with BRUE and AOP, respectively; those with LGM are 8.5 and 14.3 times more likely to have an altered PG than those with BRUE and AOP, respectively. The high risk of sleep-disordered breathing has been previously documented, especially in older children with CFA, mainly syndromic, with both mandibular and midfacial hypoplasia [16,17]. A similar situation has been reported in LGM, determining medical or surgical therapeutic behaviors with demonstrated effectiveness [17].

A team of specialists determined therapies after the PGs based on previous evaluations. Early mandibular distraction stood out in CFA with mandibular hypoplasia and supraglottoplasty in LGM in the presence of more severe alterations. In other infants, and in studies with a lower level of alteration and obstructive apnea events, prolonged treatments with oxygen therapy or non-invasive ventilation were determined initially or in the face of residual postsurgical events, continuously evaluating therapeutic results with a new PG or continuous nocturnal pulse oximetry to adjust parameters and confirm efficacy, as has been recommended in recent publications [18]. Central apneas occur mainly in premature infants due to neuro-respiratory immaturity [9]. No cases of central hypoventilation syndromes were detected.

Other specific measures were adopted in patients with altered exams to avoid recurrences and complications, such as specific medical treatments for their pathology, follow-up, and additional studies. In all of them, parents were educated on contingency aspects and safe sleeping measures [2,3,19].

Prevalence of apneas in infants with Pierre Robin sequence has been documented previously. Al-Saleh et al. studied apneas in 46 infants with a mean age of 0.8 (±0.3) y, showing a prevalence of obstructive apnea in 47% of them; 20% were described as severe apneas [20]. This prevalence is higher than in previously described normal cohorts of infants [4]. Furthermore, they found significant correlations between SpO_2_ desaturation indices and the AHI [20]. On the other hand, Poets et al. highlighted the importance of apnea diagnosis in infants with craniofacial malformations and the use of polygraphy in order to prevent possible future apneas and, therefore, a plausible development of biological rhythm abnormalities due to apneas [21].

There were some limitations in the present study that are important to consider. First, this was a retrospective sample analysis that showed the reality of apneas in newborns and small toddlers who were sent to a tertiary center in order to diagnose special conditions. It was not intended to demonstrate or validate normative values, as the sample consisted of a variety of children with underlying conditions. It was also not intended to assess polygraphic cut-off values in healthy newborns. Cardiorespiratory polygraphy was conducted, as the main objective was to assess the impact of apneas and hypopneas in these children, who had different underlying conditions. No full sleep-lab-based polysomnography was intended in this study. Nevertheless, the data obtained was interesting as it showed how different the pattern of apneas was among different diseases in children aged <3 months referred to in the sleep study.

## 4. Methods

### 4.1. Study Design and Participants

This was a retrospective, observational study with a descriptive cross-sectional analysis of baseline PG recordings in infants under three months of age. The study was conducted at Dr. Guillermo Grant Benavente Hospital, a tertiary referral center in Concepción, Chile, and included PGs performed between December 2011 and June 2023. Although data collection spanned 12 years, each recording was analyzed and reported at the time of acquisition.

Eligible participants were infants under three months of age who underwent PG due to clinical suspicion of apneas. Patients were recruited from the Pediatrics and Neonatology Units, the Neonatal Intensive Care Unit, and outpatient clinics. Only the first (baseline) PG per patient was included in the analysis.

Patients were classified according to the primary clinical indication for PG as follows:

- Infants with resolved respiratory conditions but a history of apparent life-threatening events (ALTE), later redefined as brief resolved unexplained events (BRUE), for whom PG was used as part of extended evaluation.
- Former preterm infants or neonates with episodes of oxygen desaturation—either nocturnal or during feeding—combined with abnormal overnight oximetry, in whom PG was indicated to evaluate apnea of prematurity.
- Infants with clinical suspicion of central or obstructive events due to underlying conditions such as laryngomalacia, craniofacial malformations, or neuromuscular hypotonia, whether hospitalized or referred from outpatient follow-up.

Demographic and clinical data were recorded, including gestational age at birth, corrected and postnatal age at the time of PG, weight, sex, and primary diagnosis.

### 4.2. Sleep Studies

All PG recordings were performed using the Alice PDX system (Philips Respironics, Murrysville, PA, USA) and NOX T3 (Nox Medical, Reykjavík, Iceland). The device included the following channels: nasal airflow via nasal pressure transducer, thoracic and abdominal respiratory effort (inductance bands), pulse oximetry for oxygen saturation (SpO_2_) and heart rate, and sound recording via a microphone. The equipment was installed by trained health professionals with experience in technical and procedural aspects of infant sleep studies.

Recordings were conducted overnight, starting after 10:00 PM and ending before 8:00 AM, aiming to reflect nocturnal baseline respiratory function. Infants remained under continuous nursing supervision. Parents or caregivers were present during the study and completed a structured observation sheet documenting sleep onset and termination, awakenings, feeding, vomiting, crying, vital sign checks, and sensor displacement [22].

All PGs were interpreted by the same pediatric pulmonologist trained in sleep medicine, using the prevailing clinical criteria and national or international guidelines available at that time. Interpretations were progressively updated in accordance with evolving consensus statements from the American Academy of Sleep Medicine (AASM) and consensus of the Chilean Society of Pediatric Pulmonology and the Chilean Society of Neurology, Psychiatry, and Neurosurgery [8,23].

### 4.3. Polysomnography Acceptability and Exclusion Criteria

Only PGs that met quality standards were included. Acceptability criteria were defined as follows:

- Total sleep time of at least 4 h;
- Less than 20% of the recording time affected by major artifacts or sensor disconnections across critical channels.

Exclusion criteria included the following:

- PGs not meeting the above acceptability thresholds;
- Studies conducted under supplemental oxygen or ventilatory support;
- Presence of congenital heart disease;
- Repeated PGs (only the first baseline study was analyzed).

### 4.4. Variables and Definitions

**Figure 6:**
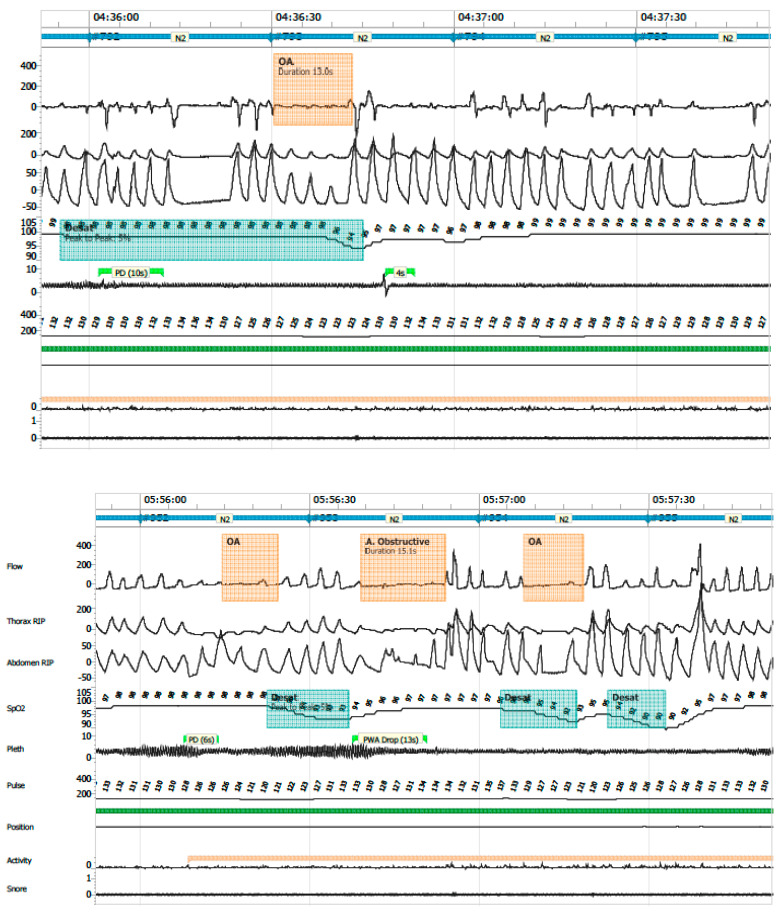
Obstructive apnea.

**Figure 7:**
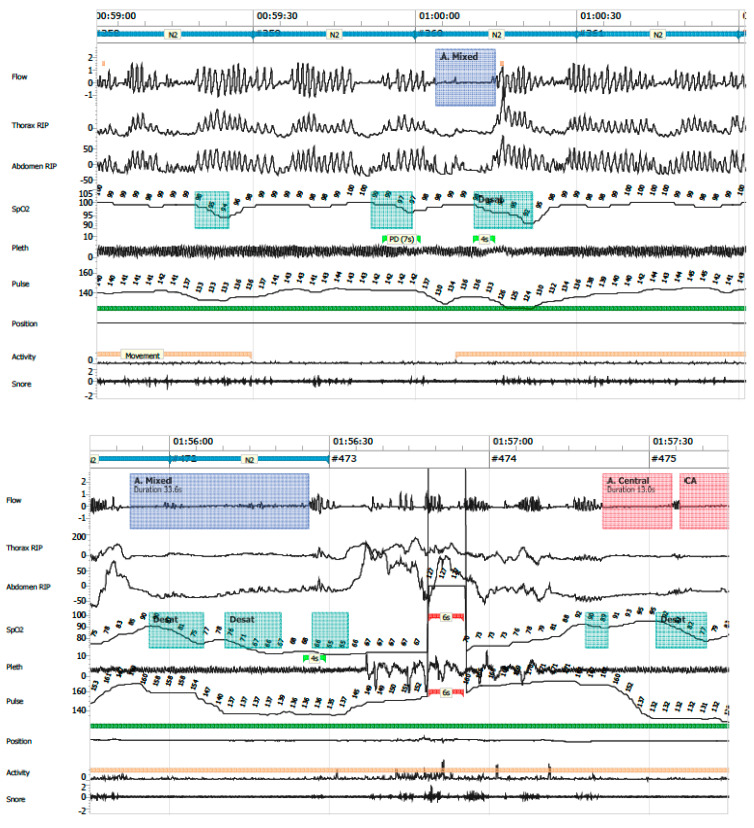
Mixed apnea.

**Figure 8:**
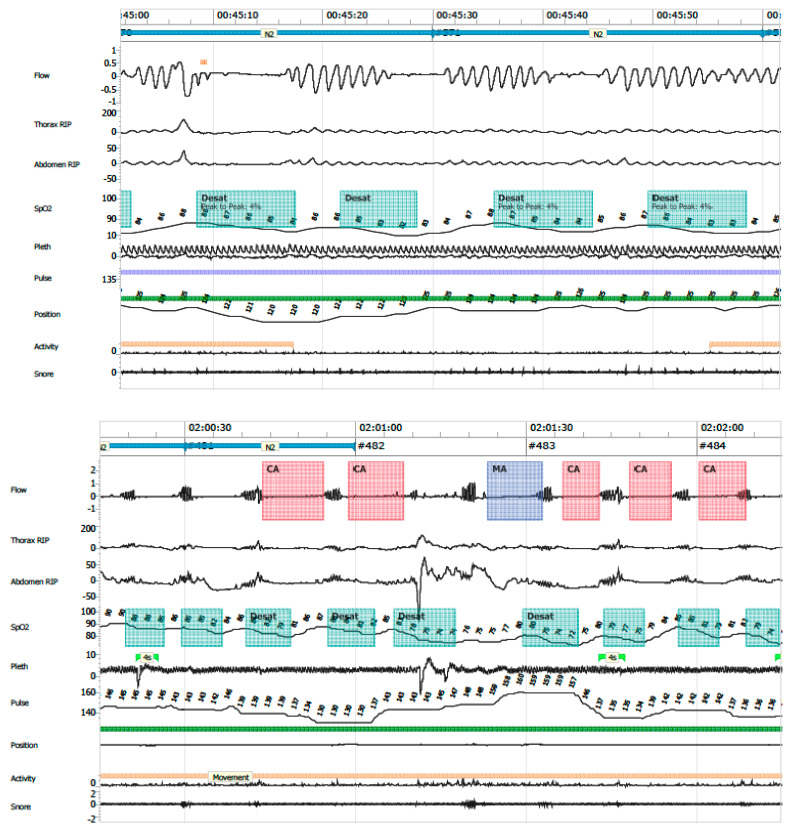
Periodic breathing.

The following PG variables were analysed:

- Apnea–hypopnea index (AHI): total number of apneas and hypopneas per hour of valid recording time.
  - Central apnea: absence or ≥90% reduction in airflow, without respiratory effort, lasting longer than the duration of two respiratory cycles.
  - Obstructive apnea: absence or ≥90% reduction in airflow with persistent respiratory effort, lasting longer than two respiratory cycles (Figure 6).
  - Mixed apneas: an apnea event with a central component (absence of respiratory effort) and transitions into an obstructive component (airflow limitation with effort) or vice versa (Figure 7).
  - Hypopnea: ≥30% reduction in nasal airflow from baseline, lasting more than the duration of two respiratory cycles, associated with a ≥3% drop in oxygen saturation.
- Oxygen desaturation index (ODI ≤ 80): number of desaturation events to ≤80% SpO_2_ per hour.
- Central apnea index (CAI): number of central apneas per hour of recording; clinical relevance was assigned to events associated with ≤80% desaturation.
- Mean SpO_2_ and time spent with SpO_2_ ≤ 90%
- Periodic breathing: defined as cyclic respiratory pattern with ≥3 successive central pauses and ≥3% desaturation (Figure 8).

Polygraphy studies were considered altered if any of the following criteria were met (8):

- AHI ≥ 5 events/hour;
- ODI ≤ 80 ≥ 1 event/hour;
- CAI ≥ 1 event/hour associated with SpO_2_ ≤ 80%;
- Mean SpO_2_ < 93%;
- Percentage of time with SpO_2_ ≤ 90% exceeding 5% of the total recording time;
- Increased periodic breathing defined as >20% of total recording time in infants younger than 37 weeks corrected gestational age, or >10% in infants older than 37 weeks corrected age.

### 4.5. Ethical Considerations

All caregivers provided written informed consent prior to the PG, which explicitly included authorization for the scientific use of anonymized results. The study protocol was approved by the Ethics Committee of the Concepción Health Service (Resolution No. 2444).

### 4.6. Statistical Analysis

The normality evaluation was performed using the Q-Q plot and the Kolmogorov–Smirnov test. Quantitative data were expressed as median and interquartile range (IQR), and qualitative data as frequency and percentage. Non-parametric statistics were performed to analyze the variables. Demographic and PG variables were compared according to diagnosis with the Kruskal–Wallis test and according to prematurity with the Wilcoxon test. The association between categorical variables, and the presence and type of alteration, was evaluated with the chi-square test, Fisher’s exact test, and chi-square with continuity correction, as appropriate. Quantitative variables were assessed using Spearman’s rho. The evaluated variables were incorporated into a binomial logistic regression model to determine the presence of PG alteration. The analysis was performed with Jamovi (v.2.3) software, considering a statistical significance of *p* < 0.05.

## 5. Conclusions

In summary, PG in infants under three months of age hospitalized and monitored for suspected apneas was feasible and allowed to confirm the presence of apneas and their level of severity to take medical or surgical measures subsequently. Studies were often abnormal in this sample and differed between diagnostic groups. Particular attention should be considered in children under three months, with CFA and LMG, since their risk is significantly higher.

### Further Research

The impact of apneas and hypopneas in infants on biological rhythm changes should be assessed in future studies. Normative values in these children seem to be difficult to obtain due to technical problems and lack of standard procedures and measurements throughout different centers. Many children are referred with already underlying conditions and diseases, like in the present study. Therefore, a comprehensive multi-center study on normative values in healthy newborns would be highly recommended.

On the other hand, as most central events and periodic breathing may probably not lead to any neurological or cardiovascular consequences, future studies should reveal the real importance of its scoring. To date, we have not found studies on the real impact of such central events on biological rhythm development or at what level, i.e., AHI, they may become a threat to future normal development of these children. Obstructive events, on the other hand, are often considered to be pathological despite the age of the patient. The present study highlighted the importance of these events in a subgroup of children referred to a tertiary center for PG. However, there is still a lack of population-based studies on the presence of obstructive sleep apneas and hypopneas in infants.

## Figures and Tables

**Table 1:** General results of the polygraphies.

Polygraphic Variables	Median	IQR	Min–Max Range
Total duration (h)	9.6	8.8–10.3	5.2–14.0
Validated duration (h)	6.7	5.9–7.9	4.0–10.5
AHI	0.5	0.1–1.7	0.0–51.1
MOAHI	0.3	0.0–1.0	0.0–49.1
CAI	0.0	0.0–0.0	0.0–17.0
OAI	0.35	0.0–0.9	0.0–49.1
DI < 80%	0.0	0.0–0.0	0.0–1.5
SpO_2_ (%)	97	96.0–98.0	90.0–99.0
Minimum SpO_2_ (%)	85	79.5–87.5	40.0–95.0
Periodic respiration (min)	3.9	0.2–12.7	0.0–179.9
Periodic respiration (%)	1.0	0.1–3.1	0.0–41.1

Respiratory indices are expressed in events/hour. The results are expressed in medians and IQR; AHI: apnea–hypopnea index; MOAHI: obstructive and mixed apnea–hypopnea index; CAI: central apnea index; DI < 80%: desaturation index less than 80%.

**Table 2:** Percentage of altered records according to established criteria.

Alteration	% (n)
AHI ≥ 5/h	50.00 [10]
SpO_2_ ≤ 90%, >5% of record	39.47 [11]
CAI ≥ 1/h with SpO_2_ ≤ 80%	23.68 [9]
Average SpO_2_ < 93	10.53 [4]
DI ≤ 80% ≥ 1/h	5.26 [2]

**Table 3:** Simple binomial logistic regression of the variables analyzed according to the presence of polygraphic alteration.

Predictor	Z	*p* Value	OR	95% Confidence Interval
Diagnosis				
CFA—AOP	3.056	0.009	21.250	2.171–207.960
HS—AOP	1.629	0.024	5.100	1.239–20.990
LGM—AOP	2.140	0.024	8.500	1.318–54.817
CFA—BRUE	3.581	0.002	35.909	3.831–336.553
HS—BRUE	2.154	0.002	8.618	2.248–33.046
LGM—BRUE	2.665	0.004	14.364	2.349–87.837

CFA: Craniofacial alterations; AOP: apnea of prematurity; LGM: laryngomalacia; HS: hypotonic syndrome.

## Data Availability

The original contributions presented in this study are included in the article/Supplementary Material. Further inquiries can be directed to the corresponding author(s).

## Supplementary Materials

The following supporting information can be downloaded at: https://www.mdpi.com/article/10.3390/clockssleep7030042/s1.
