# Supplementary material for: Polygraphic Results in High-Risk Infants Aged Under 3 Months
Source: Clocks Sleep. 2025 Aug 12;7(3):42. doi: 10.3390/clockssleep7030042 (PMC12372125; doi:10.3390/clockssleep7030042)
Supplement: Supplementary file 1 [file clockssleep-07-00042-s001.zip › clockssleep-3776878-supplementary.pdf]

Table S1. Cardiorespiratory variables obtained from polygraphy in all included patients (n=155).

ID	Gender	Age at PG (days)	AHI (events/ hours)	MOAHI (events/hours)	CAI (events/hours)	IAO (events/hours)	Periodic breathing (Min)	Periodic breathing (%)	Mean SpO2 (%)	Minimal SpO2 (%)	SpO2 <90%
1	1	17	9,8	9,8	0	9,5	7,94	2	97	79	0,2
2	1	22	9,7	9,3	0,4	7,7	54,34	16,5	96	78	1,8
3	1	71	9,5	9,5	0	9,5	0	0	94	59	1,8
4	0	38	8,4	1,8	6,6	1,8	103,81	34,5	93	62	12,4
5	0	34	8,2	8,1	0,1	7,6	1,02	0,3	98	87	0,1
6	1	7	7,5	7,5	0	7	2,69	0,6	97	87	0,7
7	1	12	6,8	6,7	0,1	6,7	5	1	96	75	0
8	0	50	6,4	6,4	0	5,9	7,54	2	94	79	1,7
9	1	56	6,3	6,3	0	5,8	0	0	98	82	0,2
10	1	26	6,3	6,3	0	4,6	15,27	3,8	93	87	1,1
11	0	71	6,2	0,6	5,6	0,6	132	40	96	43	6,2
12	0	46	51,1	49,1	2	49,1	132,41	41,1	95	73	3,4
13	1	69	4,9	4,9	0	4,9	1,72	0,4	98	91	0
14	1	54	4,5	2,4	2,1	1,5	17,9	4,4	91	72	20,2
15	0	12	4,3	4,3	0	4,3	0,7	0,1	95	78	0,9
16	0	52	4	3,9	0,1	3,3	58,49	13,4	97	78	1,4
17	0	62	31	14	17	2	82	24,9	94	40	3
18	1	59	3,5	3,5	0	2,8	7,66	2,5	96	83	1,6
19	1	92	3,4	3,4	0	3,3	5,88	1,4	97	92	0
20	1	4	3	3	0	3	9,38	2,8	96	80	0,1
21	0	13	3	3	0	2,7	38,44	10,8	95	84	1,1
22	1	49	28,9	28,9	0	28,3	0	0	97	83	1,2

23	0	11	21,1	21,1	0	20,7	0,77	0,2	97	77	1,2
24	1	15	20,7	20,7	0	20,3	0	0	97	78	0,8
25	0	32	2,6	2,6	0	2,6	29,77	7,5	95	86	0,1
26	1	53	2,5	2,5	0	2,1	0,48	0,1	97	90	0
27	0	52	2,2	1,9	0	1,9	0,81	0,2	98	89	0,01
28	0	22	2,1	0,2	1,9	0,1	89,35	17,3	96	75	6,2
29	1	38	2,1	2,1	0	1,3	24,65	8,8	98	93	0
30	1	15	19,8	19,6	0,2	16,7	33,02	10,5	96	76	1,6
31	1	84	13,4	13,4	0	13,4	0	0	96	74	1,3
32	1	64	11,5	11,5	0	10,9	0	0	96	82	1,4
33	1	23	1,9	1,9	0	1,5	62,07	14	97	82	0,5
34	1	29	1,8	1,8	0	1,8	0	0	97	87	0,1
35	1	49	1,8	1,8	0	1,6	1,28	0,4	98	88	0,2
36	1	57	1,8	1,6	0	1,6	0,46	0,1	98	85	0,1
37	1	35	1,8	9,9	1,6	0,9	3,64	0,7	94	68	11,9
38	1	84	1,7	0	0	1,7	1,5	0,3	98	89	0,1
39	1	87	1,7	0,4	1,3	0,1	179,86	35,8	95	74	5,4
40	1	40	1,7	0,1	1,6	0,1	141	25	91	51	1,6
41	1	29	1,7	1,7	0	1,7	0	0	97	88	0,3
42	0	57	1,5	1	0,5	1	11,78	3,2	98	88	1,4
43	1	49	1,5	1,5	0	1,1	0	0	98	86	0,01
44	0	26	1,4	0,7	0,7	0,7	32	5,9	97	67	0,7
45	1	82	1,3	1,3	0	0,9	0,52	0,1	98	90	0
46	0	67	1,3	1	0,3	0,9	0	0	97	84	0,1
47	0	51	1,2	1,2	0	1,2	0	0	94	80	0,01
48	1	71	1,1	1,1	0	0,9	15,3	3,8	95	86	2,6

49	1	41	1	1	0	1	9,89	2,1	98	83	1,9
50	1	54	1	1	0	0,9	34,83	10	98	89	0,1
51	1	39	1	1	0	1	9,52	1,7	98	84	0,1
52	0	30	0,9	0,9	0	0,9	0	0	98	92	0
53	1	6	0,9	0,7	0,2	0,7	4,98	1,5	96	81	3,2
54	1	12	0,9	0,9	0	0,9	0,94	0,2	95	84	0,6
55	1	44	0,9	0,7	0	0,7	15,93	6,2	98	86	0,3
56	0	11	0,9	0,6	0	0,6	3,94	1	98	87	0,3
57	0	44	0,8	0,8	0	0,8	4,47	1,1	97	87	0,2
58	0	75	0,8	0,8	0	0,8	9,56	1,8	98	89	0,01
59	0	62	0,8	0	0	0	0,54	0,2	96	83	1,9
60	1	25	0,8	0,8	0	0,6	1,6	0,4	99	92	0
61	1	26	0,7	0,7	0	0,5	22,64	5,1	98	85	0,1
62	0	90	0,7	0,7	0	0,6	0	0	98	85	0,01
63	1	49	0,7	0	0,7	0	10,19	2,9	96	74	5,9
64	1	45	0,7	0,7	0	0,7	0	0	98	81	0,4
65	0	4	0,7	0,7	0	0,5	5,58	1,6	98	79	0,2
66	1	38	0,7	0,7	0	0,3	0,91	0,2	98	89	0,01
67	1	35	0,6	0,6	0	0,6	11,2	2,7	98	84	0,5
68	1	53	0,6	0,6	0	0,6	0	0	98	87	0,1
69	1	16	0,6	0,6	0	0,6	3,12	1	98	85	0,4
70	1	7	0,6	0,6	0	0,6	94,14	22,9	98	81	0,5
71	0	22	0,6	0,1	0,5	0,1	0	0	93	74	0,6
72	1	25	0,6	0,6	0	0,4	3,57	0,9	98	81	0,01
73	1	82	0,6	0,5	0,2	0,5	4,88	1,3	94	80	3,7
74	1	85	0,5	0	0	0	2,11	0,9	97	85	1,8

75	0	65	0,5	0,5	0	0,5	2,88	0,7	98	86	0,2
76	0	42	0,5	0,1	0,4	0,1	7,17	1,4	97	78	2,5
77	1	55	0,5	0	0,5	0	8,14	2,3	97	80	1,9
78	1	66	0,5	0,5	0	0,4	2,31	0,5	97	87	0,8
79	1	47	0,5	0	0,4	0,2	7,64	2,1	98	85	0,7
80	0	81	0,5	0,5	0	0,5	2,93	0,8	98	92	0
81	1	39	0,5	0,5	0	0,2	1,9	0,4	96	87	0,2
82	0	88	0,5	0,5	0	0,5	0,54	0,1	96	87	0,8
83	1	50	0,5	0	0	0,4	34,42	6,9	94	82	0,9
84	1	81	0,5	0,2	0,3	0,2	0	0	94	70	0,5
85	1	4	0,4	0,4	0	0,4	1,16	0,3	98	85	1
86	0	31	0,4	0,4	0	0,4	36,34	7,6	98	90	0,1
87	1	59	0,4	0,4	0	0,4	6,91	2,1	98	89	0,2
88	1	13	0,4	0,4	0	0,4	7,77	1,7	94	87	1,4
89	1	10	0,4	0,4	0	0,4	43,97	10,5	96	87	0,5
90	0	17	0,4	0	0,4	0	45	15	94	60	0,4
91	1	85	0,4	0,4	0	0,4	1,02	0,2	98	85	0,3
92	1	17	0,4	0,4	0	0,4	0,6	0,1	97	90	0
93	0	28	0,4	0,4	0	0,3	32,81	7,1	96	84	0,4
94	1	48	0,4	0,4	0	0,2	0	0	98	88	0,5
95	0	41	0,4	0,4	0	0,4	4,8	1,1	96	73	2,9
96	0	93	0,4	0	0,4	0	15,2	3,8	98	69	3
97	0	80	0,37	0,12	0,25	0,12	0	0	96	77	0,37
98	1	37	0,3	0,3	0	0,3	15,97	4,4	97	87	0,2
99	0	8	0,3	0,3	0	0,1	4,11	1	97	84	0
100	1	68	0,3	0,3	0	0	0,59	0,3	98	87	0,2

101	0	89	0,3	0,3	0	0	4,68	1,2	98	87	0,3
102	1	59	0,3	0	0	0,3	0	0	98	88	0,2
103	1	22	0,3	0,3	0	0,3	25,52	5,3	98	88	0,01
104	1	58	0,3	0	0	0,1	4,97	1,2	99	88	0
105	0	37	0,3	0,2	0,1	0,1	12,51	3	93	78	11,3
106	0	84	0,2	0,2	0	0,2	0,63	0,3	90	80	25,4
107	1	15	0,2	0,2	0	0,2	35,58	9,6	94	83	2,2
108	0	19	0,2	0,2	0	0,2	33,21	8,5	98	86	0
109	1	13	0,2	0,3	0	0,3	96,29	22,4	98	88	0,2
110	1	75	0,2	0,1	0,1	0,1	0	0	98	78	0,2
111	1	41	0,2	0	0,2	0	15,76	2,9	96	78	1,7
112	1	92	0,2	0,2	0	0	15,3	3,8	95	70	3
113	1	3	0,1	0	0,1	0	8	1,5	96	80	0,1
114	0	37	0,1	0,1	0	0	6,26	1,1	97	84	0,7
115	1	46	0,1	0,1	0	0,1	20	3,9	97	77	0,4
116	1	16	0,1	0,1	0	0,1	1,02	0,2	99	88	0
117	0	40	0,1	0,1	0	0,1	0	0	97	89	0,1
118	0	22	0,1	0	0	0,2	5,88	2,4	98	87	0,1
119	1	35	0,1	0	0	0	35,45	8,1	97	78	1,3
120	1	5	0,1	0	0,1	0	6,41	1,5	97	79	1,2
121	0	30	0	0	0	0	4,15	0,9	97	82	0,2
122	1	55	0	0	0	0	0	0	99	95	0
123	1	63	0	0	0	0	5,56	1,4	99	82	0,5
124	1	52	0	0	0	0	0	0	97	82	0
125	1	47	0	0	0	0	0	0	97	84	0
126	1	54	0	0	0	0	28,04	4,8	97	88	0,01

127	1	33	0	0	0	0	0	0	99	85	0
128	1	49	0	0	0	0	2,19	0,5	94	85	4,2
129	0	64	0	0	0	0	2	0,4	95	86	0
130	1	82	0	0	0	0	1,23	0,4	98	83	0,7
131	1	29	0	0	0	0	0	0	99	87	0
132	0	59	0	0	0	0	20,43	5	98	87	1,3
133	0	26	0	0	0	0	5	1	97	88	0
134	1	12	0	0	0	0	10,35	2,2	97	89	0,1
135	1	13	0	0	0	0	9,6	1,6	97	88	0
136	1	38	0	0	0	0	0	0	96	59	0
137	1	68	0	0	0	0	25	7,6	98	86	0
138	1	56	0	0	0	0	12,95	4	98	85	0,1
139	1	15	0	0	0	0	0,92	0,2	98	88	0,1
140	1	30	0	0	0	0	0	0	99	92	0
141	1	12	0	0	0	0	0	0	94	87	1
142	0	73	0	0	0	0	0	0	98	84	0
143	0	43	0	0	0	0	0	0	98	88	0,9
144	0	62	0	0	0	0	11,95	2,4	96	82	0,4
145	1	29	0	0	0	0	2	0,5	96	89	0
146	1	46	0	0	0	0	0	0	97	84	0
147	0	41	0	0	0	0	0	0	99	92	0
148	1	62	0	0	0	0	0	0	99	93	0,3
149	0	22	0	0	0	0	0	0	97	86	0
150	1	6	0	0	0	0	3	0,5	97	86	0
151	0	92	0	0	0	0	9,07	2,9	96	82	1,2
152	0	9	0	0	0	0	2	0,3	98	92	0

153	1	46	0	0	0	0	0	0	92	79	17,9
154	0	38	0	0	0	0	0	0	96	91	0
155	0	10	0	0	0	0	33,23	8,5	97	84	0,2
